# Assessment Scales in Cerebral Palsy: A Comprehensive Review of Tools and Applications

**DOI:** 10.7759/cureus.47939

**Published:** 2023-10-30

**Authors:** Chaitanya Kumar Javvaji, Jayant D Vagha, Revat J Meshram, Amar Taksande

**Affiliations:** 1 Pediatrics, Jawaharlal Nehru Medical College, Datta Meghe Institute of Higher Education and Research, Wardha, IND

**Keywords:** interdisciplinary collaboration, personalized medicine, emerging trends, assessment scales, motor dysfunction, cerebral palsy assessment

## Abstract

Cerebral palsy (CP) is a complex neurological condition characterized by motor dysfunction affecting millions worldwide. This comprehensive review delves into the critical role of assessment in managing CP. Beginning with exploring its definition and background, we elucidate the diverse objectives of CP assessment, ranging from diagnosis and goal setting to research and epidemiology. We examine standard assessment scales and tools, discuss the challenges inherent in CP assessment, and highlight emerging trends, including integrating technology, personalized medicine, and neuroimaging. The applications of CP assessment in clinical diagnosis, treatment planning, research, and education are underscored. Recommendations for the future encompass standardization, interdisciplinary collaboration, research priorities, and professional training. In conclusion, we emphasize the importance of assessment as a compass guiding the care of individuals with CP, issuing a call to action for improved assessment practices to shape a brighter future for those affected by this condition.

## Introduction and background

Cerebral palsy (CP) is a complex and heterogeneous group of non-progressive, lifelong motor disorders originating from early brain development and characterized by varying degrees of movement dysfunction. CP is one of the most prevalent childhood neurological conditions globally, affecting approximately two to three per 1,000 live births. It presents a significant challenge to affected individuals, their families, and healthcare providers due to its wide-ranging impact on motor function, communication, and daily life [1-3].

The term “cerebral palsy” encompasses a spectrum of motor impairments, manifesting as spasticity, dyskinesia, ataxia, or a combination thereof. These motor disturbances result from brain damage or abnormal brain development before, during, or shortly after birth. While CP is non-progressive, its clinical presentation can evolve due to growth and aging. Understanding the nuances of this condition is vital for effective management and intervention [4].

Assessment plays a pivotal role in the management of individuals with CP. It serves as the foundation upon which healthcare professionals, educators, and caregivers develop tailored interventions, monitor progress, and make informed decisions about treatment strategies. Assessment in CP extends beyond mere diagnosis; it encompasses the comprehensive evaluation of an individual's motor abilities, communication skills, cognitive function, and overall quality of life [5].

The multidimensional nature of CP necessitates a systematic and standardized approach to assessment. Precise and objective assessment tools enable clinicians to characterize an individual's condition accurately, identify specific areas of impairment, and track changes over time. Moreover, assessments provide the basis for setting realistic goals and evaluating the effectiveness of therapeutic interventions. In essence, assessment empowers healthcare teams to provide targeted, evidence-based care that enhances individuals with CP's well-being and functional independence [6].

This review aims to provide a comprehensive examination of the assessment scales and tools used to evaluate individuals with CP. We will delve into various aspects of CP assessment, including the most employed assessment scales, their applications in clinical practice, and the emerging trends and challenges within this field. By synthesizing existing knowledge and highlighting recent developments, this review aims to assist healthcare professionals, researchers, and educators in making informed decisions regarding selecting and implementing assessment tools for individuals with CP.

## Review

Background on CP

Etiology and Causes of CP

CP, a complex neurological condition, can arise from many factors, broadly categorized into four distinct groups. First, prenatal factors encompass a range of conditions and exposures during pregnancy that may contribute to the development of CP. These include maternal infections, exposure to harmful toxins or drugs, and genetic factors that may increase the risk of brain abnormalities in the developing fetus. Second, perinatal factors occurring around birth are another critical dimension [7]. Events such as birth asphyxia, premature birth, low birth weight, and complications during delivery can significantly increase the likelihood of CP. Third, postnatal factors come into play when brain injuries occur after birth [8]. These injuries can result from head trauma, infections, strokes, or other neurological insults, further highlighting the multifactorial nature of CP causation. Finally, and perhaps most perplexing, are the cases where the exact etiology of CP remains unknown. These instances underscore the complexity of the condition, suggesting that a combination of factors or genetic predispositions may interplay to contribute to the development of CP [8].

Prevalence and Demographics

Global prevalence: CP is a notable global prevalence, affecting approximately two to three per 1,000 live births worldwide. However, it is essential to recognize that prevalence rates vary considerably between countries and regions. These disparities are attributed to a complex interplay of factors, including differences in healthcare access, the quality of perinatal care, and socioeconomic conditions. Regions with limited access to advanced medical care may have higher rates of CP due to challenges in preventing and managing risk factors during pregnancy and birth [9].

Age of diagnosis: The diagnosis of CP typically occurs in early childhood, often within the first two years of a child's life. This early diagnosis is prompted by recognizing motor development delays, as children with CP may exhibit distinctive signs such as delayed crawling, walking, or reaching developmental milestones. Early diagnosis is crucial as it facilitates timely interventions and access to support services that significantly improve outcomes [3].

Gender: CP does not discriminate by gender, affecting both males and females. While there is a slight predominance of CP in males, this gender difference is generally not considered significant. CP is an inclusive condition, and its impact varies widely among affected individuals, irrespective of gender. Therefore, comprehensive assessment and support should be provided to all individuals with CP, regardless of gender [10].

Socioeconomic factors: Socioeconomic factors play a substantial role in the risk and prevalence of CP. Children born into disadvantaged socio-economic backgrounds may face a higher risk of CP due to various challenges, including limited access to quality prenatal care, healthcare services, and nutritional support. Socioeconomic disparities can impact the early identification and management of risk factors during pregnancy and childbirth. Therefore, addressing these disparities and improving healthcare access for vulnerable populations is essential in reducing the burden of CP and ensuring equitable care for all children [11].

Types and Classification of CP

Spastic CP: Spastic CP is the most prevalent form, affecting approximately 70%-80% of individuals with CP. It is characterized by increased muscle stiffness and tone, resulting in stiff and sometimes jerky movements. The severity of spastic CP can vary widely, and it may affect specific limbs (monoplegia), one side of the body (hemiplegia), or all four limbs (quadriplegia). Spasticity can lead to mobility challenges, contractures, and difficulties in fine motor control [12].

Dyskinetic CP: Dyskinetic CP is characterized by involuntary and uncontrolled movements ranging from slow and writhing (athetoid) to repetitive and spasmodic (dystonic). Individuals with dyskinetic CP often struggle to control their posture and coordinated movements. These uncontrollable movements can make everyday activities, such as feeding and communication, challenging and exhausting [13].

Ataxic CP: Ataxic CP is distinguished by poor coordination, balance, and shaky, imprecise movements. Individuals with ataxic CP may struggle with tasks requiring fine motor skills, such as writing or buttoning a shirt. Their gait may appear unsteady, with a wide-based stance, making walking and balance challenging [14].

Mixed CP: In some cases, individuals exhibit a combination of features from the types, leading to a diagnosis of mixed CP. This can present unique challenges as individuals may experience complex symptoms, including spasticity, dyskinesia, and ataxia. The specific manifestations and severity vary widely among individuals with mixed CP [15].

Assessment in CP

Objectives of Assessment

Diagnosis: One of CP assessment's primary and foundational objectives is facilitating an accurate diagnosis. Early diagnosis, often within the first two years of life, is pivotal as it allows for timely and targeted interventions and support. This ensures that individuals with CP receive the care they need as soon as possible, increasing the potential for improved outcomes [16].

Characterization of impairments: CP assessment goes beyond diagnosis to provide a detailed characterization of impairments in various domains. It offers valuable insights into the nature and extent of motor function limitations, communication challenges, cognitive impairments, and other associated difficulties. This comprehensive understanding forms the basis for individualized treatment planning [17].

Goal setting: Assessment results provide a baseline for setting achievable and meaningful goals for individuals with CP. These goals are tailored to address specific needs and aspirations related to mobility, communication, self-care, or other aspects of daily life. Establishing clear goals helps guide therapeutic interventions and monitor progress [18].

Monitoring progress: Over time, assessment tools serve as valuable instruments for monitoring an individual's progress. They enable healthcare professionals to track changes in functional abilities, assess the impact of interventions, and make necessary adjustments to treatment plans. This ongoing assessment is essential for optimizing outcomes and ensuring that interventions remain aligned with evolving needs [19].

Research and epidemiology: Beyond clinical applications, assessment data are pivotal for research and epidemiological studies on CP. These data aid scientists and researchers in understanding the prevalence causes, risk factors, and long-term outcomes of CP. Researchers can make informed advancements in the field by collecting and analyzing assessment data, ultimately leading to more effective assessment and treatment strategies [20].

Challenges in CP Assessment

Heterogeneity of CP: CP is marked by a significant degree of heterogeneity, with a broad spectrum of motor impairments, associated disabilities, and varying levels of cognitive function. This inherent diversity poses a challenge when developing assessment tools. A one-size-fits-all approach may not capture individuals' nuanced needs and abilities with CP. Therefore, assessment tools must be flexible and adaptable to accommodate the unique profiles of each person [1].

Communication difficulties: Communication difficulties are common among individuals with CP, affecting their ability to participate in traditional assessment methods. Many individuals may have limited verbal communication skills, necessitating the consideration of alternative communication strategies. Augmentative and alternative communication (AAC) tools and techniques, such as communication boards or devices, should be integrated into the assessment process to ensure that individuals with CP can effectively express their needs and preferences [21].

Aging and changing needs: As individuals with CP progress through different stages of life, their needs and abilities may change significantly. Assessment must be an ongoing and dynamic process that adapts to these evolving requirements. Pediatric assessments may focus on developmental milestones, while assessments for adults may emphasize functional independence and quality of life. Regular re-evaluation ensures that interventions remain aligned with the changing needs of individuals with CP [22].

Inter-rater reliability: Achieving consistent assessment results can be challenging due to potential variations in the interpretation of assessment tools by different healthcare professionals. Maintaining high inter-rater reliability is crucial to ensure that assessment outcomes are accurate and reliable. This can be achieved through standardized training, calibration exercises, and ongoing collaboration among healthcare providers to align assessment practices [23].

Importance of Standardized Assessment Tools

Objective measurement: Standardized assessments provide a structured and objective framework for measuring an individual's abilities and limitations. By relying on predefined criteria and scoring systems, these assessments reduce the potential for subjective bias, ensuring that the evaluation process remains impartial and consistent. This objectivity is critical in CP assessment, where precise and unbiased measurements are essential for accurate diagnosis and intervention planning [24].

Comparability: Standardized assessment tools enable comparing results across individuals and settings. This comparability is invaluable for both research and clinical decision-making. Healthcare providers can benchmark an individual's performance against established norms and reference data, facilitating a better understanding of their strengths and areas requiring intervention. Additionally, researchers can analyze and aggregate data from diverse populations, enhancing our collective knowledge of CP [25].

Baseline data: Standardized assessments provide a baseline against which an individual's progress can be tracked over time. By conducting assessments at regular intervals, healthcare professionals can identify changes in motor function, communication abilities, and other domains. This longitudinal data is essential for evaluating the effectiveness of interventions and making informed adjustments to treatment plans. It empowers healthcare providers to tailor interventions to each individual's evolving needs, optimizing outcomes [26].

Evidence-based care: Standardized assessments often align with evidence-based care guidelines and recommendations. This alignment ensures that individuals with CP receive treatments, therapies, and interventions rigorously tested and proven effective through research and clinical trials. By adhering to evidence-based practices, healthcare providers can confidently deliver care grounded in the latest scientific knowledge, increasing the likelihood of positive outcomes and improved quality of life for individuals with CP [27].

Overview of Assessment Domains

Motor function: Motor function assessments focus on evaluating an individual's physical abilities, including mobility, muscle strength, coordination, and the capacity to perform activities of daily living. These assessments help healthcare providers and therapists understand the extent of motor impairments and formulate targeted interventions to enhance mobility and functional independence. Prominent tools in this domain include the Gross Motor Function Classification System (GMFCS) and the Manual Ability Classification System (MACS) [28].

Communication: Communication assessments are centered around an individual's ability to express themselves and comprehend others. Communication can be particularly challenging for individuals with CP, and these assessments aid in classifying an individual's communication abilities and identifying areas that require support or intervention. The Communication Function Classification System (CFCS) is an example of a tool used to categorize communication skills [17].

Cognition: Cognitive assessments are crucial for evaluating an individual's intellectual abilities, memory, problem-solving skills, and overall cognitive development. These assessments have significant implications for educational planning and intervention strategies. Educators and therapists can tailor instruction and support to maximize learning potential by understanding an individual's cognitive strengths and areas of difficulty [29].

Sensory function: Some individuals with CP may experience sensory impairments, such as visual or auditory deficits. Assessments in this domain aim to identify sensory challenges and assess their impact on daily life. Understanding sensory function helps adapt interventions and create environments that accommodate sensory needs [30].

Psychosocial and emotional well-being: Assessments in the psychosocial and emotional domains are essential to evaluate an individual's mental and emotional well-being. Individuals with CP may face unique psychosocial challenges related to self-esteem, coping, and social integration. Identifying psychosocial needs is critical for providing appropriate support and resources to promote emotional well-being and resilience [31].

Common assessment scales and tools

Gross Motor Function Classification System

Description and scoring: The GMFCS is a valuable and widely utilized tool in assessing gross motor function in children and adolescents with CP [32]. This system provides a structured framework for classifying an individual's functional abilities into five distinct levels, each reflecting a different degree of mobility and independence:

Level I: Individuals classified at this level can walk without significant limitations. While they may occasionally require assistive mobility devices for extended distances or specific situations, their primary mode of mobility is independent walking [32].

Level II: Those falling into this category can walk but do so with limitations. They often rely on mobility aids such as crutches, canes, or walkers to support their ambulation. While they may have some independence in walking, they benefit from assistive devices [32].

Level III: Individuals at Level III walk with assistance, typically from a caregiver or using assistive devices. While they can take steps, they may rely on a wheelchair for mobility over longer distances or when managing daily activities [32].

Level IV: At this level, individuals primarily rely on a power wheelchair for mobility. However, they may have the ability to stand with assistance. Their mobility is mainly dependent on powered mobility devices [32].

Level V: Level V represents the most severe limitations in gross motor function. Individuals classified at this level typically rely entirely on a power wheelchair for mobility. Significant additional impairments often impact their daily life and functional independence [32]. Scoring in the GMFCS is based on an individual's self-initiated movement and mobility, with higher levels indicating more significant functional limitations.

Applications and Clinical Use

Treatment planning: One of the primary clinical applications of the GMFCS is treatment planning. Healthcare professionals, including clinicians, therapists, and rehabilitation specialists, rely on the GMFCS to assess an individual's gross motor function and mobility. By classifying individuals into the appropriate GMFCS level, healthcare teams can tailor treatment strategies and interventions to match an individual's specific functional abilities. This personalized approach ensures that therapy and interventions are practical and realistic, aligning with the individual's current motor skills and needs [33].

Prognosis: The GMFCS is valuable for motor development and functional independence prognoses. By assessing an individual's gross motor function, clinicians can offer insights into an individual's likely motor progression and potential for achieving greater independence in activities of daily living. This information is invaluable for families and caregivers, as it helps set realistic expectations and provides a basis for goal setting and intervention planning [34].

Research and epidemiology: Beyond its clinical applications, the GMFCS is a crucial asset in research and epidemiological studies related to CP. Researchers use the GMFCS classification to categorize and compare the functional abilities of individuals with CP across different populations, settings, and time frames. This standardized classification system enables researchers to gather valuable data on the distribution of gross motor function in CP, contributing to our understanding of the condition's prevalence, trends, and outcomes. This, in turn, informs future developments in assessment, treatment, and care for individuals with CP [35].

Manual Ability Classification System

Description and scoring: The MACS is a valuable assessment tool designed to evaluate the manual abilities and hand function of individuals with CP [36]. This classification system categorizes individuals into five distinct levels based on their manual abilities.

Level I: Individuals classified at Level I can effectively handle objects with their hands. They demonstrate high manual dexterity and can efficiently perform various daily activities [36].

Level II: Those falling into Level II can manage most objects but may experience limitations regarding the quality and speed of their hand movements. While they can perform various tasks, their manual abilities are less efficient than those at Level I [36].

Level III: Individuals at Level III can handle some objects but often encounter difficulty doing so. Many individuals in this category require assistance or adaptive devices to complete manual tasks effectively [36].

Level IV: Level IV represents a significant limitation in handling objects. Even with assistance, individuals at this level have minimal capacity for independent manual activities. They may require substantial support to complete tasks [36].

Level V: The most severe limitations in manual abilities are found at Level V. Individuals classified here typically cannot handle objects and may have minimal or no active use of their hands. They often rely on alternative methods and assistive technology for daily tasks [36].

Applications and Clinical Use

Treatment planning: The MACS plays a central role in treatment planning for individuals with CP. Therapists and healthcare professionals rely on MACS classifications to gain insights into an individual's manual abilities and limitations. This information informs the selection of appropriate therapeutic interventions and strategies to improve hand function and manual dexterity. By tailoring treatment plans to an individual's specific MACS level, therapists can design realistic, effective, and targeted interventions to address their unique needs [37].

Assessment of progress: One of the primary clinical uses of the MACS is to monitor an individual's progress over time. By assessing changes in manual abilities, therapists and healthcare teams can evaluate the effectiveness of therapeutic interventions and rehabilitation programs. This ongoing assessment ensures that interventions align with the individual's evolving needs and functional improvements. Tracking progress through MACS classifications enables healthcare providers to make data-driven adjustments to treatment plans and celebrate milestones achieved by individuals with CP [38].

Design of assistive devices: The MACS classification system is instrumental in designing and customizing assistive devices and adaptive technology. By understanding an individual's manual abilities and limitations, engineers and assistive technology specialists can develop devices that enhance an individual's independence in activities of daily living. These devices are tailored to the specific needs and capabilities of the individual, maximizing their functional autonomy and quality of life [39].

Communication Function Classification System

Description and scoring: Level I: Individuals at Level I effectively use all forms of communication, including speech and AAC methods. They can express themselves comprehensively and switch between different communication modalities [40].

Level II: Those classified at Level II primarily use speech as their primary mode of communication. However, they may occasionally require AAC support in specific situations or for more complex communication needs [40].

Level III: Individuals at Level III rely on speech and AAC to communicate effectively. While they can use speech, they rely more on AAC systems and strategies for successful communication [40].

Level IV: Level IV represents individuals who primarily use AAC for communication, with occasional attempts at speech. While they may still try to use speech, their primary means of communication is through AAC systems [40].

Level V: The most severe communication limitations are observed at Level V. Individuals in this category rely entirely on AAC methods for communication and have limited or no speech abilities [40].

Applications and Clinical Use

Communication intervention: The CFCS is a valuable guide for clinicians and speech-language therapists in developing and implementing communication intervention strategies. By classifying individuals into different CFCS levels, therapists gain insights into an individual's current communication abilities and needs. This information forms the foundation for designing tailored interventions that align with the individual's communication profile. Whether it involves speech therapy, AAC training, or other communication-focused interventions, the CFCS helps ensure that strategies are customized to meet the individual's unique requirements [41].

Selection of AAC systems: The CFCS is critical in determining the most appropriate AAC system for an individual with CP. AAC encompasses a range of tools and methods, including communication boards, speech-generating devices, and sign language. By assessing an individual's communication abilities and reliance on different modalities, the CFCS aids in making informed decisions about selecting and customizing AAC systems. This ensures that individuals with CP can access communication tools that best match their needs and preferences, enhancing their ability to express themselves effectively [42].

Outcome measurement: The CFCS is a valuable tool for assessing the effectiveness of communication interventions and tracking improvements in communication skills over time. By periodically reassessing an individual's communication abilities and comparing them to previous CFCS classifications, clinicians can evaluate the impact of interventions and adjust strategies as needed. This dynamic assessment process ensures that communication interventions remain relevant and effective, supporting individuals with CP to achieve communication goals and enhance their quality of life [43].

Pediatric Evaluation of Disability Inventory (PEDI)

Description and scoring: Self-Care - This domain assesses an individual's capacity to perform essential self-care activities independently. Self-care activities include feeding, dressing, grooming, and other personal hygiene and daily living activities. The PEDI evaluates an individual's level of independence or dependence in these self-care activities [44].

Mobility: The mobility domain evaluates an individual's mobility and transportation skills. It encompasses a range of abilities, including sitting, crawling, standing, walking, and other forms of locomotion. Through this domain, the PEDI assesses an individual's mobility limitations and the support or assistance required to move effectively [45].

Social function: The social function domain focuses on an individual's interaction with others and their participation in social and recreational activities. It assesses how well an individual engages with peers, family members, and the community. This domain also considers an individual's involvement in activities such as playing, participating in group activities, and interacting with others in various social settings [46].

Applications and Clinical Use

Treatment planning: The PEDI is essential for healthcare professionals, therapists, and educators caring for children and adolescents with disabilities. It assists in identifying areas of functional impairment and provides a comprehensive assessment of an individual's functional strengths and limitations. This information serves as a foundation for developing targeted and individualized intervention plans. Healthcare teams can optimize the effectiveness of therapeutic strategies, rehabilitation programs, and educational interventions by tailoring interventions to address specific areas of need identified through the PEDI assessment [47].

Outcome measurement: The PEDI is a valuable instrument for tracking changes in functional abilities over time. It offers a standardized framework for assessing an individual's functional progress and measuring the impact of various interventions and therapies. By periodically reassessing an individual's performance using the PEDI, healthcare professionals can objectively evaluate the effectiveness of treatments and make informed decisions about intervention modifications. This dynamic assessment process also enables setting achievable goals for improvement, motivating individuals with disabilities to work toward greater functional independence and participation in daily life [48].

Research and epidemiology: The PEDI is a valuable tool in research studies and epidemiological investigations focused on children and adolescents with disabilities, including those with CP. It enables researchers to gather quantitative data on this population's functional abilities and limitations. By employing the PEDI in research, investigators can contribute to a better understanding of the functional profiles of individuals with disabilities, identify trends and patterns, and explore factors influencing functional outcomes. This research, in turn, informs the development of evidence-based practices and policies aimed at improving the lives of children and adolescents with disabilities [49].

Other Relevant Assessment Scales

The WeeFIM (functional independence measure for children): The WeeFIM is a tool designed to evaluate self-care, mobility, and cognition in children with disabilities, including those with CP. It assesses a child's level of independence in various daily activities, helping healthcare professionals and therapists tailor interventions and support services to enhance functional independence and quality of life [50].

The Vineland adaptive behavior scales: The Vineland Scales are widely used to measure adaptive behavior and daily living skills in individuals with developmental and intellectual disabilities. These scales assess an individual's ability to perform age-appropriate tasks and adapt to their environment. They provide valuable insights into an individual's adaptive functioning and are often used in assessing individuals with CP to guide intervention planning [51].

The Bayley Scales of infant and toddler development: The Bayley Scales are a well-established tool for assessing infants and toddlers. They assess various domains, including cognitive, motor, and language development. The Bayley Scales are valuable for identifying developmental delays and providing early intervention for infants and toddlers at risk for or diagnosed with CP. Early assessment and intervention are crucial for optimizing developmental outcomes in this population [52].

Emerging trends in CP assessment

Technological Advancements

Use of wearable devices: Continuous monitoring - Wearable devices enable continuous monitoring of motor function and movement patterns throughout the day. Unlike traditional assessments that capture only isolated snapshots of an individual's abilities, these devices provide a continuous data stream. This continuous monitoring offers a more complete and nuanced understanding of an individual's daily activities, mobility, and functional abilities. It allows healthcare professionals to track changes over time and observe variations in motor function under different conditions and activities [53].

Objective data: Wearable devices provide objective and quantifiable data, reducing the potential for observer bias that may be present in traditional assessments. The data collected by these devices are based on real-time measurements, ensuring that assessments are based on accurate and reliable information. This objectivity enhances the validity and precision of the assessment process, supporting more informed clinical decisions and intervention planning [54].

Remote monitoring: The integration of wearable devices facilitates remote monitoring of individuals with CP, offering numerous benefits. Telehealth consultations can be enhanced by wearable technology to assess an individual's motor function and progress remotely. This is particularly valuable in overcoming geographical barriers and ensuring that individuals in underserved or remote areas access specialized care and assessment services. Remote monitoring also allows for frequent check-ins, adjustments to treatment plans, and timely interventions, all of which contribute to improved outcomes and quality of life for individuals with CP [55].

Longitudinal data: Wearable devices generate longitudinal data that can be analyzed over time. This longitudinal approach enables healthcare professionals to identify trends, track progress, and assess the effectiveness of interventions and treatments. Longitudinal data can also be valuable in research studies, contributing to a deeper understanding of the natural history of CP and the impact of various interventions on motor function and daily life [56].

Telehealth Assessment

Remote consultations: Telehealth enables healthcare professionals to conduct assessments remotely through video conferencing and other digital platforms. This approach allows individuals with CP and their families to connect with specialized healthcare providers without needing in-person visits. Remote consultations are valuable for follow-up assessments, progress monitoring, and ongoing care management. They offer a convenient and efficient means of conducting assessments while minimizing the need for extensive travel and in-person appointments [57].

Accessibility: Telehealth assessments increase the accessibility of specialized care and assessments for individuals with CP, especially those in rural or underserved areas. In regions where access to healthcare providers with expertise in CP may be limited, telehealth bridges the geographical gap. This expanded access to assessment services ensures that individuals in remote or underserved communities receive timely and high-quality care, ultimately improving their outcomes [58].

Family-centered care: Telehealth assessments can promote family-centered care by actively involving family members and caregivers in the assessment process. Family members can participate in remote consultations, share valuable information about the individual's daily life, and provide insights into their functional abilities and challenges. This collaborative approach ensures that the assessment considers the holistic needs and goals of the individual with CP, considering their unique family dynamics and support systems [59].

Continuity of care: Telehealth assessments support continuity of care by facilitating ongoing communication between healthcare providers and individuals with CP. Regular remote consultations allow for frequent check-ins, progress tracking, and adjustments to treatment plans as needed. This continuous care model ensures that individuals receive consistent and coordinated support, improving outcomes and quality of life [57].

Reduced barriers: Telehealth assessments can help reduce barriers to care, such as transportation limitations, mobility challenges, and scheduling conflicts. By eliminating the need for physical travel and offering flexible scheduling options, telehealth makes assessments more accessible and convenient for individuals and their families [60].

Personalized Medicine and Treatment Planning

Genetic testing: Genetic testing is pivotal in identifying specific genetic factors associated with CP. These genetic insights can contribute to a more precise diagnosis and a deeper understanding of the underlying causes of CP in individual cases. With genetic information, healthcare professionals can tailor interventions and therapies to address specific genetic factors. For example, if a genetic mutation is identified, targeted therapies or interventions can be explored to mitigate its effects or address related complications. Genetic testing also supports genetic counseling, enabling families to make informed decisions about family planning and potential genetic risks [61].

Pharmacogenomics: Pharmacogenomics, a component of personalized medicine, considers an individual's genetic makeup when prescribing medications. For individuals with CP, this approach can be precious. Healthcare providers can identify potential genetic factors influencing medication metabolism and response by analyzing an individual's genetic profile. This personalized approach reduces the risk of adverse drug reactions and ensures that medications are tailored to the individual's genetic predispositions. It optimizes treatment outcomes and minimizes the need for trial-and-error approaches to medication management [62].

Precision therapies: Emerging precision therapies, including stem cell-based treatments, hold promise in targeting the underlying genetic and cellular factors associated with CP. Stem cell therapies, for example, aim to repair or regenerate damaged brain tissue, potentially offering significant benefits to individuals with CP. Precision medicine enhances the likelihood of successful outcomes by customizing these therapies based on an individual's genetic characteristics and specific CP subtypes. These innovative therapies align to address the root causes of CP and promote neurological repair and functional improvement [63].

Integration of Neuroimaging and Biomarkers

Neuroimaging: Advanced neuroimaging techniques, such as functional MRI (fMRI) and diffusion tensor imaging (DTI), offer detailed insights into the structural and functional aspects of the brain. These modalities allow healthcare professionals to visualize brain regions, connectivity patterns, and areas of injury or dysfunction associated with CP. Neuroimaging enhances diagnostic accuracy by identifying specific brain abnormalities and their spatial distribution and provides a foundation for tailored treatment planning. Additionally, neuroimaging aids in understanding the evolving nature of brain injuries in CP, informing decisions about interventions and therapeutic strategies [64].

Biomarkers: Biomarkers found in blood-cerebrospinal fluid or obtained from imaging data are being explored as potential indicators of CP. Researchers are investigating the presence and characteristics of biomarkers associated with the condition. These biomarkers have the potential to facilitate early diagnosis, offer insights into the underlying mechanisms of CP, predict prognosis, and assess treatment responses. Finding reliable biomarkers could revolutionize CP assessment by providing objective and quantifiable measures complementing clinical evaluations [65].

Early detection: The integration of neuroimaging and biomarkers promises to enable earlier detection of CP. By identifying subtle brain changes and biomarker patterns indicative of CP risk or onset, healthcare professionals can intervene at an earlier stage. Early detection allows timely and targeted interventions, therapies, and support services. This proactive approach is crucial for maximizing the developmental outcomes and quality of life of individuals with CP, as early interventions can address motor and cognitive challenges during critical developmental periods [66].

Applications of CP assessment

Clinical Diagnosis

Early identification: Assessment tools are instrumental in the early identification of CP, often within the first two years of life. This early diagnosis is essential because it allows healthcare professionals to recognize signs of CP and initiate timely interventions. Early intervention and therapy can significantly improve an individual's developmental outcomes, motor function, and overall quality of life. Assessment tools serve as a vital component of early screening and diagnostic processes, enabling the identification of CP at its earliest stages [67].

Timely intervention: Early diagnosis facilitated by assessment tools allows prompt access to appropriate interventions and services. Once CP is identified, healthcare professionals can develop tailored treatment plans that address the specific needs and challenges of the individual. These interventions may include physical, occupational, speech, and assistive devices. Timely intervention optimizes developmental progress, mitigates functional limitations, and enhances the individual's long-term prospects [68].

Classification and tailored treatment: Assessment scales, such as the GMFCS and the MACS, are valuable tools for classifying the type and severity of CP. This classification helps healthcare professionals understand the individual's functional abilities and limitations across motor and manual domains. The classification informs treatment planning by providing a framework for selecting appropriate interventions and therapies. Tailored treatment plans are essential because they address the specific challenges and goals of everyone, ensuring that interventions are both practical and personalized to their unique needs [32].

Prognosis

Predicting developmental trajectories: Assessment results offer insights into an individual's functional abilities and challenges. These findings, combined with the knowledge of CP's natural history and typical developmental patterns, enable healthcare providers to make informed predictions about an individual's developmental trajectory. By understanding where an individual is in their developmental journey, healthcare providers can anticipate potential challenges, milestones, and areas of improvement. This predictive information is valuable for setting appropriate treatment goals, tailoring interventions, and preparing families for the journey [6].

Setting realistic expectations: Prognostic information derived from assessments helps set realistic expectations for functional improvement and long-term outcomes. By sharing assessment findings and prognoses with families and individuals with CP, healthcare providers foster informed decision-making and empower families to make choices that align with their goals and values. Realistic expectations also play a crucial role in managing the emotional and psychological aspects of living with CP, providing a framework for understanding the challenges and successes encountered [69].

Treatment Planning and Intervention

Individualized treatment: Assessment data play a central role in developing individualized treatment plans for individuals with CP. By thoroughly assessing an individual's functional abilities, challenges, and specific needs across domains such as motor function, communication, and cognitive development, healthcare professionals can create treatment plans that are precisely tailored to the individual. These plans include targeted interventions and therapies designed to address the unique characteristics and goals of each person with CP. Individualized treatment plans maximize the effectiveness of care by focusing on the areas that require attention, leading to better outcomes and improved quality of life [70].

Goal setting: Assessment results provide a baseline for setting achievable and meaningful goals for therapy and intervention. These goals are essential for motivating individuals with CP and their families, as they offer a clear sense of direction and purpose. By establishing specific, measurable, and time-bound goals based on assessment findings, healthcare providers can track progress and celebrate milestones along the way. Goal setting encourages individuals to engage in rehabilitation actively and empowers them to work toward their full potential [71].

Monitoring progress: Regular assessments are a fundamental component of ongoing care for individuals with CP. They serve as a means of monitoring an individual's response to therapy and interventions over time. By conducting assessments at regular intervals, healthcare professionals can objectively measure progress, identify areas of improvement, and detect any emerging challenges. The data from these assessments guide decisions about treatment plan continuation, modification, or refinement. Monitoring progress through assessments ensures that interventions remain aligned with the individual's evolving needs and goals, ultimately optimizing outcomes and functional independence [72].

Research and Epidemiological Studies

Understanding prevalence: Assessment tools are essential components of epidemiological studies aimed at estimating the prevalence of CP within specific populations. Researchers can identify and classify individuals with CP within a defined sample by systematically applying assessment scales and criteria. This prevalence data is invaluable for public health planning and resource allocation. It informs healthcare systems, policymakers, and organizations about the burden of CP in a given region or population, guiding the development of targeted healthcare services, early intervention programs, and support networks [73].

Identifying risk factors: Research studies that incorporate CP assessments contribute to the identification of potential risk factors and etiological factors associated with the condition. Through comprehensive assessments and data analysis, researchers can explore demographic, genetic, environmental, and prenatal factors that may contribute to the development of CP. This information is crucial for expanding our understanding of CP's causes and risk factors, which, in turn, can inform preventive strategies and early intervention efforts [74].

Evaluating interventions: Clinical trials and research studies leverage assessment data to rigorously evaluate the effectiveness of interventions, therapeutic modalities, and emerging treatments for CP. Researchers use standardized assessment tools to measure the impact of interventions on various domains, including motor function, communication, and quality of life. The data generated from these assessments provide empirical evidence of the benefits and outcomes associated with different interventions. This evidence-based approach helps guide treatment decisions, refine therapeutic approaches, and advance the field of CP care by identifying interventions that significantly improve functional outcomes [75].

Education and Early Intervention Programs

Individualized education plans (IEPs): Assessment results are the foundation for developing IEPs for children with CP. These plans are highly personalized, considering each child's strengths, challenges, and needs. Assessment data inform the selection of appropriate educational goals, accommodations, and support services tailored to the child's unique requirements. IEPs ensure that children with CP receive a customized and inclusive education that maximizes their learning potential [76].

Early intervention programs: Assessments are fundamental in early intervention programs for infants and toddlers with CP. Early identification of developmental delays or challenges is crucial, as it allows for prompt access to specialized services and therapies. Assessment tools help professionals identify areas of developmental concern and tailor interventions to address them. Early intervention programs, supported by assessment data, aim to optimize developmental outcomes and prepare children with CP for a successful transition into educational settings [77].

Progress monitoring: Ongoing assessments conducted within educational settings are essential for monitoring a child's progress. Educators and therapists use assessment data to track a child's developmental milestones, academic achievements, and functional abilities. Regular assessments provide insights into the effectiveness of teaching strategies and interventions. Suppose a child with CP requires additional support or adjustments to their educational plan. In that case, progress monitoring helps educators make informed decisions and adapt their approaches to meet the child's evolving needs [78].

Resource allocation: Assessment data are pivotal in resource allocation and funding decisions for educational and early intervention programs. By providing objective information about the unique needs of children with CP, assessment results help education authorities and policymakers allocate resources appropriately. These resources may include funding for specialized services, assistive technology, and professional development for educators. Effective resource allocation ensures that individuals with CP receive the support and accommodations necessary for a quality education [79].

Future directions and recommendations

Standardization and Consistency in CP Assessment

Inter-rater reliability improvement: Efforts should be made to improve inter-rater reliability among healthcare professionals conducting CP assessments. This can be achieved through standardized training programs and calibration exercises. These initiatives help ensure that different assessors interpret and apply assessment tools consistently, reducing variations in assessment results. Collaboration among healthcare institutions and professional organizations can facilitate the development and dissemination of training resources to enhance inter-rater reliability [23].

Global standardization: Collaborative efforts should aim to standardize assessment tools and diagnostic criteria for CP. Establishing internationally recognized standards promotes consistency in diagnosis and treatment planning across regions and cultures. Global standardization facilitates cross-cultural comparisons and ensures that individuals with CP receive consistent and evidence-based care regardless of where they live. International organizations and experts in the field can play a pivotal role in driving this standardization process [80].

Continual updates: Assessment scales and tools should be regularly updated to reflect the latest research findings and clinical best practices. The field of CP assessment is dynamic, with advancements in diagnostic techniques and interventions. Researchers, clinicians, and experts should collaborate to incorporate new insights and evidence into assessment protocols to maintain the relevance and accuracy of assessment tools. This includes refining existing tools and developing new ones that capture the multidimensional nature of CP [20].

Interdisciplinary collaboration: Collaboration among healthcare professionals, including neurologists, orthopedic surgeons, therapists, psychologists, and educators, is essential for comprehensive CP assessments. A multidisciplinary approach ensures that assessments encompass all relevant domains, including motor function, communication, cognition, and psychosocial well-being. Collaborative teams can provide a holistic understanding of an individual's needs and design comprehensive care plans that address all aspects of living with CP [81].

Family-centered assessment: Involving families and caregivers in the assessment process is essential. They possess valuable insights into an individual's daily life, challenges, and progress. Including family perspectives in assessments fosters a family-centered approach to care, where families actively participate in decision-making and goal-setting. Healthcare professionals should actively seek family input and consider their unique perspectives when conducting assessments [82].

User-friendly technology: Developing user-friendly assessment technology and tools can streamline the assessment process and improve data accuracy. Incorporating technology, such as digital apps and wearable devices, can enhance the efficiency of assessments and reduce the burden on both assessors and individuals with CP. User-friendly interfaces and digital platforms can make assessments more accessible and inclusive [83].

Education and training: Continuous education and training programs for healthcare professionals involved in CP assessment are vital. These programs should update assessors on the latest assessment protocols, research findings, and best practices. Additionally, training programs should emphasize the importance of cultural competence and sensitivity, ensuring that assessments are culturally responsive and consider diverse backgrounds and experiences [84].

Integration of Assessment Data into Care Plans

Interdisciplinary collaboration: Encourage and facilitate interdisciplinary collaboration among healthcare providers, therapists, educators, and caregivers. This collaboration ensures that assessment data are seamlessly integrated into comprehensive care plans that address all aspects of an individual's well-being. Effective communication and teamwork among professionals from various disciplines promote holistic and coordinated care for individuals with CP [85].

Electronic health records (EHRs): Develop and implement EHR systems that capture, store, and share assessment data efficiently and securely. EHRs enable healthcare providers across different settings to access and contribute to a centralized repository of information. This ensures that assessment data are readily available for decision-making, treatment planning, and ongoing monitoring. EHRs also support the continuity of care as individuals transition between healthcare providers and facilities [86].

Patient-centered care: Promote a patient-centered approach involving individuals with CP and their families in assessing and developing care plans. This approach recognizes the individual's and family's expertise and preferences, making them active partners in decision-making. When formulating care plans, healthcare providers should engage in open and collaborative discussions with individuals and their families, considering their goals, values, and priorities. This patient-centered approach ensures that care plans are tailored to each individual's unique needs and aspirations with CP [87].

Care coordination: Establish mechanisms for effective care coordination to ensure that assessment data are translated into actionable care plans that are consistently implemented across different healthcare settings. Care coordinators or case managers can be central in facilitating communication among healthcare providers, therapists, educators, and families. They help ensure that assessment findings are translated into interventions, therapies, and educational strategies that align with the individual's goals and needs [88].

Education and training: Provide education and training for healthcare providers, therapists, educators, and caregivers on integrating assessment data into care plans. Training programs should emphasize the practical application of assessment results in treatment planning and intervention strategies. Healthcare professionals and caregivers should have the knowledge and skills to interpret assessment data accurately and translate them into meaningful care plans [89].

Data accessibility: Ensure assessment data are easily accessible to individuals with CP and their families. This may involve providing individuals and families access to their health records and assessment results through secure online portals or communication channels. Empowering individuals and families with access to their data fosters transparency and active involvement in their care [90].

Quality assurance: Implement quality assurance measures to monitor and improve the integration of assessment data into care plans. Regular audits, peer reviews, and feedback mechanisms can help identify areas for improvement in the assessment-to-care planning process. Continuous quality improvement efforts ensure that assessment data contribute effectively to positive outcomes for individuals with CP [91].

Research Priorities in CP Assessment

Early detection biomarkers: Invest in research to identify reliable biomarkers for the early detection of CP. Early intervention is crucial for improving outcomes in individuals with CP. Biomarkers, such as genetic markers, neuroimaging findings, or biochemical indicators, can facilitate early diagnosis, allowing timely access to interventions and support. Research efforts should aim to develop and validate biomarker-based screening and diagnostic tools that are sensitive, specific, and accessible [67].

Advanced neuroimaging: Continue to advance neuroimaging techniques, including fMRI, DTI, and other modalities, to gain a deeper understanding of the underlying neurological mechanisms in CP. Research in this area can provide valuable insights into brain structure, connectivity, and function, helping to identify areas of injury, neural plasticity, and potential targets for therapeutic interventions. Collaboration among neuroimaging researchers and clinicians is essential to translate neuroimaging findings into clinical practice [92].

Effectiveness of emerging technologies: Research to evaluate the effectiveness of emerging technologies, such as wearable devices and telehealth, in improving CP assessment and care. Wearable devices offer the potential for continuous monitoring of motor function and daily activities, providing valuable data for assessment and intervention planning. Telehealth has the potential to overcome geographical barriers, making assessments and interventions more accessible to individuals with CP in underserved areas. Rigorous research studies should assess these technologies' accuracy, feasibility, and cost-effectiveness in real-world clinical settings [93].

Long-term outcomes: Prioritize longitudinal studies to assess interventions' long-term outcomes and impact based on assessment data. Understanding the trajectory of individuals with CP over their lifespan is critical for tailoring care plans and optimizing interventions. Longitudinal research should investigate factors influencing functional outcomes, quality of life, and societal participation. It should also explore the role of early interventions in shaping long-term outcomes and consider the diverse needs of individuals with different types and severities of CP [94].

Genomic and pharmacogenomic research: Continue genomic and pharmacogenomic research to identify genetic factors associated with CP and to personalize treatment approaches. Genetic testing can help identify at-risk individuals for CP and inform tailored interventions. Pharmacogenomics considers an individual's genetic makeup when prescribing medications, reducing the risk of adverse effects and optimizing treatment outcomes. Research in this area should focus on identifying genetic markers linked to CP and developing pharmacogenomic guidelines for medication management [95].

Functional outcome measures: Develop and validate functional outcome measures that capture the multidimensional nature of CP. Assessment tools should encompass motor function, communication, cognition, psychosocial well-being, and quality of life. Research should prioritize the refinement of existing assessment scales and the development of new tools that reflect the diverse and evolving needs of individuals with CP [70].

Global collaboration: Foster global collaboration and data sharing among researchers, clinicians, and organizations involved in CP assessment and care. International collaboration facilitates the pooling of diverse datasets, enhances research findings' generalizability, and accelerates field progress. Collaborative networks can support large-scale research studies, meta-analyses, and the development of international guidelines for CP assessment and management [96].

Training and Education for Professionals

Specialized training: Provide healthcare professionals involved in CP assessment with specialized training in using assessment tools and interpreting results. This training should emphasize the importance of standardized assessment protocols, calibration exercises, and techniques for improving inter-rater reliability. Training programs should be designed to ensure that assessors have the necessary skills and knowledge to administer assessments accurately and consistently [97].

Cultural competency: Incorporate cultural and sensitivity training into CP assessment education. Healthcare professionals and educators should be equipped to address the diverse needs of individuals with CP and their families, recognizing the influence of cultural factors on healthcare decisions and preferences. Training should emphasize the importance of culturally responsive care and communication [98].

Continuing education: Promote ongoing education and professional development opportunities for healthcare providers, therapists, educators, and other professionals involved in CP assessment and care. Continuing education programs should cover the latest advancements in CP assessment, diagnostic criteria, treatment modalities, and best practices. Professionals should be encouraged to stay updated on research findings and evidence-based guidelines to maintain current knowledge [99].

Patient and family education: Ensure that individuals with CP and their families are educated about the assessment process, the significance of assessment results, and their role in care planning. Education should empower individuals and families to participate in decision-making and goal-setting actively. Healthcare providers and educators should engage in open and transparent communication to explain assessment findings, treatment options, and the potential impact on an individual's daily life [100].

Accessible resources: Make educational resources on CP assessment accessible to healthcare professionals, educators, individuals with CP, and their families. This may include digital resources, pamphlets, online courses, and support groups. Accessible resources facilitate self-directed learning and provide a valuable reference for individuals and families seeking information about CP assessment and care [101].

Interdisciplinary collaboration: Encourage interdisciplinary collaboration among professionals from various fields, including neurology, rehabilitation medicine, therapy, and education. Collaborative team meetings and case discussions promote knowledge sharing and a holistic approach to CP assessment and care. Interdisciplinary collaboration ensures that assessment data are effectively translated into comprehensive care plans that address all aspects of an individual's well-being [102].

Feedback mechanisms: Establish feedback mechanisms that allow healthcare professionals and educators to provide input on the effectiveness of training programs and educational resources. Continuous improvement processes should be implemented to refine training curricula based on participant feedback. Regular assessments of training outcomes can help identify areas for enhancement and tailor educational initiatives to meet the evolving needs of the healthcare workforce [103].

Peer learning and mentoring: Encourage peer learning and mentoring opportunities within the healthcare and education communities. Experienced professionals can serve as mentors to guide newer practitioners in CP assessment. Peer support networks can facilitate the exchange of knowledge and practical insights, fostering professional growth and competence [104].

## Conclusions

In conclusion, CP assessment is not merely a clinical process; it is the cornerstone of compassionate care and the gateway to a better quality of life for those with this condition. Throughout this review, we have illuminated the multifaceted role of assessment, from its diagnostic significance to its pivotal role in personalized treatment plans, research endeavors, and educational support programs. We have underscored the challenges accompanying this critical task and explored promising technological advancements and interdisciplinary collaborations shaping the future of CP assessment. As we look ahead, we recognize the imperative for standardized practices, seamless integration of assessment data into care plans, and sustained research efforts to unlock new possibilities. Our collective responsibility, as caregivers, researchers, and advocates, is to heed this call to action, fostering a world where individuals with CP are empowered by the accuracy and compassion of their assessments and where their potential knows no bounds.
